# Supplementary material for: Factors associated with international humanitarian aid appeal for disasters from 1995 to 2015: A retrospective database study
Source: PLoS One. 2023 Jun 1;18(6):e0286472. doi: 10.1371/journal.pone.0286472 (PMC10234527; doi:10.1371/journal.pone.0286472)
Supplement: Attachment — Submitted filename: Response to Reviewers PLOS ONE R&R.docx [file pone.0286472.s002.docx]

**Reviewer #1:**

1. *Page 6, line 139- 141; legend of figure is inserted into the body of manuscript.*

Thank you for pointing this out. Following PLOS ONE guidelines, (<https://journals.plos.org/plosone/s/figures>), we have removed Figure label, title, and legends from the TIFF file, and kept these in the manuscript text in read order.

2. *Page 14, line 275-277; the number (1)~(3) are confusing with the number for references*

Thank you for pointing this out. We have removed the numbered points in the prose to avoid confusion.

**Reviewer #2:**

3. *Discuss these points further:
   Dependence: One of the most significant disadvantages of international aid is that it can create a culture of dependence in recipient countries. If countries become reliant on foreign aid, they may not develop their own economies, which can hinder long-term growth and development.

   Corruption: International aid can also lead to corruption, as governments or organizations may use the funds for their own purposes rather than the intended recipients. This can prevent aid from reaching those who need it most and may even exacerbate the problems it was intended to solve.

   Disruption of Local Markets: International aid can disrupt local markets by flooding them with cheap or free goods, which can harm local businesses and prevent economic growth.

   Conditionality: Many countries that provide international aid may attach conditions to it, such as requiring the recipient country to adopt specific economic policies or make political changes. This can limit a country's sovereignty and prevent it from making its own decisions.

   Unsustainable Solutions: Aid programs may be designed to address immediate needs, such as providing food and medicine during a crisis. However, they may not be sustainable in the long term and may not address the root causes of poverty or underdevelopment.

   My own thought is that aid should be stopped. I wish this paper had made this recommendation. It is disempowering and manipulative whether in disaster or not. It is actually immoral for one country to claim to help another. There is no disabled country on earth, give them a chance to evolve and grow.*

Thank you for these valued opinions. We share the Reviewer’s concern against uninvited, unregulated, and intrusive international humanitarian action. This shared mission is in fact the motivation for this project. As a scientific manuscript, we base our discussion on available data and attempt to best balance our opinions with the understandably varied views and experiences of the international community. To bolster our discussion with the Reviewer’s valued opinions, we added a forerunning paragraph to the effect of the Reviewer’s comments by referencing PAHO and WHO publications.

4. *Inclusion/exclusion criteria: While the inclusion/exclusion criteria are clearly stated, it may be helpful to provide some justification for why these specific criteria were chosen. For example, why was the threshold for human deaths set at 10? Why was the year 1995 chosen as the starting point for the search? and why stopped 2015.*

Thank you for evaluating this point. A literature search and subject expertise does not yield specific justification for the database author’s inclusion criteria, although it is widely accepted. We added discussion on this as a limitation and referenced a recent article which arrived at the same conclusion. We added elaboration in the methods section on the rationale behind limiting the search until 2015. The rationale for 1995 as a start point is also elaborated in the methods section.

5. *Missing data: The authors acknowledge that missing data was present in the dataset, but it is not clear how they handled this issue. While the use of multiple imputation is mentioned, it is not clear how this method was implemented or what software was used.*

Thank you for requesting clarification. We added the specifications for the multiple imputation into the methods section in the main text: (Fully conditional specification, 10 iterations, predictive mean matching, 10 imputations, SPSS 26.0).

6. *Logistic regression: The authors state that logistic regression was used to examine the independent association between factors and international aid appeal, but it is not clear what specific factors were included in the regression model. Providing a list of these factors would help readers understand the analysis more clearly.*

Thank you for requesting clarification. We added underlines to all variables in Table 1 which were entered into logistic regression. We also added a sentence in the legend of Table 1: Significant p-values were underlined and the corresponding variables were entered in the logistic regression.

7. *Ethics approval: While the authors state that ethics approval was not required, it may be helpful to provide some additional information on the ethical considerations related to the use of publicly available data, particularly given the sensitive nature of the topic (i.e. disasters and deaths).*

Thank you for requesting additional information. We elaborated in the methods section on the primary reasons that ethics approval was not required, and made special effort to address potentially sensitive factors like deaths and injury. Our main justification is the non-individual patient level data, and the publicly available data source.
